# The American Medical Association

**Published:** 1888-05

**Authors:** 


American Medical Association.—The officers elect of the American Medical Association are as follows:

President—W. W. Dawson, of Cincinnati.

First Vice President—W. L. Schenck, of Kansas.

Second Vice President—Frank Woodbury, of Pennsylvania.

Third Vice President—H. O. Walker, of Michigan.

Fourth Vice President—J. W. Bailey, of Georgia.

Treasurer—R. J. Dunglison, of Pennsylvania.

Secretary—W. B. Atkinson, of Pennsylvania.

Librarian—C. H. A. Kleinschmidt, Washington, D. C.

Trustees—E. M. Moore, of New York; John H. Hallister, of Illinois; Joseph M. Toner, District of Columbia.

Judicial Council—W. A. Phillips, of Kansas; A. M. Pollock, of Pennsylvania; U. C. Vanbibber, of Maryland; J. A. Hibbard, of Indiana; Chas. S. Wood, of New York; J. McGaston, of Georgia; W. H. O. Taylor, of New York; Geo. J. Porter, of Connecticut.

Newport, Rhode Island, was chosen for the next place of meeting in June of next year.

